# A single point in protein trafficking by *Plasmodium falciparum* determines the expression of major antigens on the surface of infected erythrocytes targeted by human antibodies

**DOI:** 10.1007/s00018-016-2267-1

**Published:** 2016-05-19

**Authors:** Jo-Anne Chan, Katherine B. Howell, Christine Langer, Alexander G. Maier, Wina Hasang, Stephen J. Rogerson, Michaela Petter, Joanne Chesson, Danielle I. Stanisic, Michael F. Duffy, Brian M. Cooke, Peter M. Siba, Ivo Mueller, Peter C. Bull, Kevin Marsh, Freya J.I. Fowkes, James G. Beeson

**Affiliations:** 1 Burnet Institute for Medical Research and Public Health, 85 Commercial Road, Melbourne, VIC 3001 Australia; 2 Walter and Eliza Hall Institute of Medical Research, Parkville, VIC Australia; 3 Department of Medical Biology, University of Melbourne, Parkville, VIC Australia; 4 Department of Medicine, University of Melbourne, Parkville, VIC Australia; 5 Melbourne School of Public Health, University of Melbourne, Parkville, VIC Australia; 6 Research School of Biology, Australian National University, Canberra, ACT Australia; 7 Institute for Glycomics, Griffith University, Southport, QLD Australia; 8 Papua New Guinea Institute of Medical Research, Madang, Papua New Guinea; 9 Centre for Geographic Medicine Research, Coast, Kenya Medical Research Institute, Kilifi, Kenya; 10 Programs in Infection and Immunity and Cardiovascular Disease, Monash Biomedicine Discovery Institute, Monash University, Melbourne, VIC Australia; 11 Department of Epidemiology and Preventive Medicine and Department of Infectious Diseases, Monash University, Melbourne, VIC Australia; 12 Department of Microbiology, Monash University, Melbourne, VIC Australia

**Keywords:** Malaria, Immunity, Vaccines, Antibodies, Trafficking, *Plasmodium falciparum*

## Abstract

Antibodies to blood-stage antigens of *Plasmodium falciparum* play a pivotal role in human immunity to malaria. During parasite development, multiple proteins are trafficked from the intracellular parasite to the surface of *P. falciparum*-infected erythrocytes (IEs). However, the relative importance of different proteins as targets of acquired antibodies, and key pathways involved in trafficking major antigens remain to be clearly defined. We quantified antibodies to surface antigens among children, adults, and pregnant women from different malaria-exposed regions. We quantified the importance of antigens as antibody targets using genetically engineered *P. falciparum* with modified surface antigen expression. Genetic deletion of the trafficking protein skeleton-binding protein-1 (SBP1), which is involved in trafficking the surface antigen PfEMP1, led to a dramatic reduction in antibody recognition of IEs and the ability of human antibodies to promote opsonic phagocytosis of IEs, a key mechanism of parasite clearance. The great majority of antibody epitopes on the IE surface were SBP1-dependent. This was demonstrated using parasite isolates with different genetic or phenotypic backgrounds, and among antibodies from children, adults, and pregnant women in different populations. Comparisons of antibody reactivity to parasite isolates with SBP1 deletion or inhibited PfEMP1 expression suggest that PfEMP1 is the dominant target of acquired human antibodies, and that other *P. falciparum* IE surface proteins are minor targets. These results establish SBP1 as part of a critical pathway for the trafficking of major surface antigens targeted by human immunity, and have key implications for vaccine development, and quantifying immunity in populations.

**Electronic supplementary material:**

The online version of this article (doi:10.1007/s00018-016-2267-1) contains supplementary material, which is available to authorized users.

## Introduction

Malaria caused by *Plasmodium falciparum* remains a major cause of morbidity and mortality worldwide [1]. Clinical symptoms result from the blood-stage replication of the parasite, which multiplies within erythrocytes. After erythrocyte invasion by the merozoite form, the intraerythrocytic parasite undergoes development through ring, trophozoite and schizont stages before rupturing to release merozoites to perpetuate the asexual cycle. *P. falciparum*-induced modifications of infected erythrocytes (IEs) (reviewed in [2, 3]) include the appearance of knob structures on the IE membrane, created by the deposition of knob-associated histidine-rich protein (KAHRP) molecules on the cytoplasmic face of the erythrocyte membrane. These knob structures enable the adhesion of IEs to vascular receptors and sequestration of IEs to prevent clearance by the spleen. *Plasmodium falciparum* erythrocyte membrane protein 1 (PfEMP1), which is presented on the IE surface by the knob structures, is a key ligand for IE sequestration in the vasculature [4], and is not found other human malaria species. A central feature in the pathogenesis of *P. falciparum* malaria is the sequestration of IEs in the vasculature of various organs, which is not prominent in other human malarias such as *P. vivax, P. knowlesi, P. ovale, P. malariae*.

*Plasmodium falciparum* IE surface antigens are important targets of acquired antibodies that contribute to immunity and function by opsonizing IEs for phagocytic clearance by monocytes, macrophages and other cells, and by inhibiting vascular adhesion and rosette formation (reviewed in [5]). These antigens are polymorphic, and antibodies that are acquired after exposure to infection show a degree of strain specificity [6, 7]. Antibodies to IE surface antigens have been associated with protection in longitudinal studies (reviewed in [5]) and effective immunity is thought to require the acquisition of a broad repertoire of antibodies to different *P. falciparum* strains or variants [6, 8]. Efforts to define the nature and importance of IE surface antigens and to quantify which antigens are targeted by naturally acquired immunity have been challenging due to the many IE surface proteins identified, the complexity of multigene families encoding these antigens, and limited tools available. In addition to PfEMP1, other antigens identified on the IE surface include repetitive interspersed family proteins (RIFIN) [9], subtelomeric variable open reading frame proteins (STEVOR) [10–12], surface-associated interspersed gene family proteins (SURFIN) [13, 14] and others. These proteins are encoded by multigene families with variation in expression between isolates, which has presented many challenges to studying them as immune targets. Recent approaches to study immune responses using genetically modified parasites, in a Kenyan coastal population, identified PfEMP1 as a major target of acquired human antibodies [15]. However, the relative importance of PfEMP1 and the significance of other IE surface proteins remain unclear.

The trafficking of antigens to the IE surface is only partly understood, and further detailed knowledge is important for defining and quantifying key targets of protective immunity. Since erythrocytes are devoid of protein trafficking machinery and *de novo* protein synthesis, *P. falciparum* has to synthesize and export parasite proteins for trafficking, as well as for parasite development (reviewed in [16]). Several *P. falciparum* proteins are exported into the host erythrocyte to support the correct trafficking and surface display of proteins in the erythrocyte membrane (reviewed in [2, 5]). The export of PfEMP1, and other IE surface proteins, is highly complex due to their large size, the number of membranes that must be traversed before reaching the IE surface and the involvement of various chaperone proteins (reviewed in [16–18]). Skeleton-binding protein 1 (SBP1) [19] is an important protein of membranous structures in the IE cytosol called Maurer’s clefts (MC) and is required for the loading of PfEMP1 molecules into the MC [20, 21]. Although the role of SBP1 in trafficking PfEMP1 has been established, its role in trafficking other IE surface antigens or membrane-bound proteins is unclear. Orthologues of SBP1 have not yet been reported in other *Plasmodium* species causing human malaria, but has been identified in the chimpanzee malaria, *P. reichenowi,* which is a close relative of *P. falciparum* and also expresses PfEMP1 orthologues [20–22]. This suggests that SBP1 has evolved to export PfEMP1 and possibly other IE surface antigens in *P. falciparum*, and its relative, *P. reichenowi* [20, 21]. Transcription of *pfsbp1* commences shortly after erythrocyte invasion and persists throughout parasite development [19]. SBP1 is a type 2 integral membrane protein (48 kDa) with the N-terminal domain buried within the MC and the C-terminus exposed within the IE cytoplasm where it interacts with erythrocyte proteins such as LANCL1, spectrin and protein 4.1 [19, 23, 24].

In this study, we aimed to quantify the importance of SBP1 in the trafficking of IE surface antigens that are important targets of protective immunity in *P. falciparum* and evaluate the importance of PfEMP1 and other IE surface antigens as targets of naturally acquired antibodies, including functional antibodies that mediate the opsonic phagocytosis of IEs, which is an important mechanism for clearance of infection [25, 26]. These aims were achieved by using parasites with a targeted deletion of *pfsbp1* or genetically engineered to inhibit *var* gene and PfEMP1 expression and evaluating antibodies to parental parasites compared to genetically modified parasites. We applied these tools to human studies in Kenya, Malawi and Papua New Guinea (PNG), and included serum samples from children, adults and pregnant women to represent the spectrum of malaria exposure and immunity observed globally.

## Results

### Generation and characterization of *P. falciparum* with SBP1 deletion

**Fig. 1:**
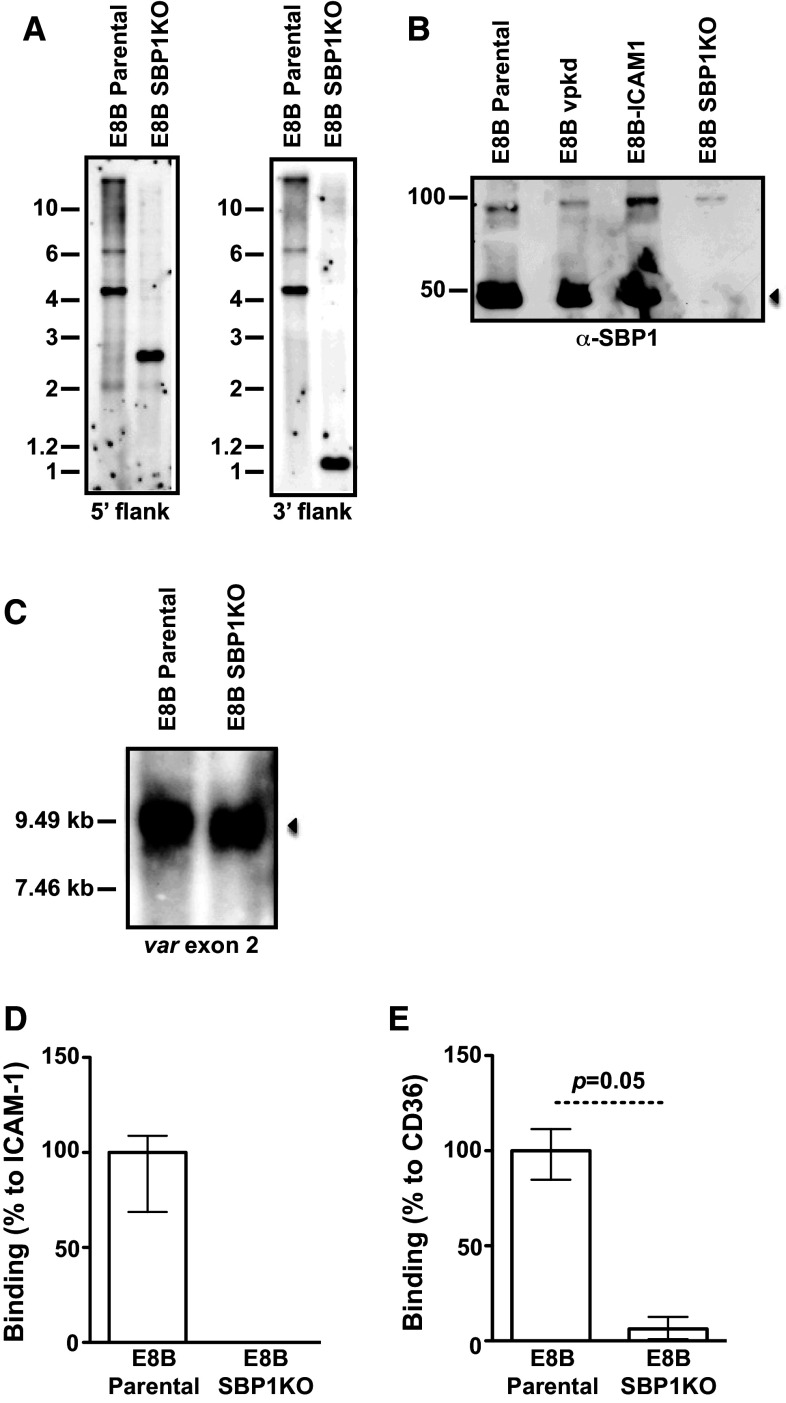
Phenotypic analyses of *pfsbp1* knock-out (SBP1KO) parasites. **a** Southern blot analysis of the *pfsbp1* locus in E8B parental and E8B SBP1KO parasites. Genomic DNA was digested with *Eco*RI/*Bgl II* and probed with the 5′ or 3′ targeting sequence. Predicted sizes for the plasmid were 6.9 kb (for both 5′ and 3′ probes) and 4.6 kb for E8B parental (for both 5′ and 3′ probes); predicted sizes for E8B SBP1KO were 2.7 kb (for 5′ probe) and 1.1 kb (for 3′ probe). **b** Western blot analyses of membrane extracts from mature trophozoite IEs probed with α-SBP1 antibodies. Antibodies detected SBP1 at approximately 50 kDa (*arrow*) in E8B parental, E8Bvpkd (PfEMP1 knock-down) and E8B-ICAM1 (parental line selected on ICAM1), which was absent in E8B SBP1KO. The band at 100 kDa present in all lanes corresponds to antibody cross reactivity. **c** Northern blot analysis of *var* gene transcription by hybridization with a specific *var* exon 2 sequence. The position of molecular weight standards (kb) is indicated on the *left* and the *arrow* represents *var* transcripts. In both E8B parental and E8B SBP1KO parasites, similar *var* transcripts were detected. RNA was extracted from highly synchronous ring stage IEs at approximately 10 h post-invasion. Adhesion of IEs to immobilized ICAM-1 (**d**) and CD36 (**e**) was substantially reduced in E8B SBP1KO compared to E8B parental. Values are expressed as a percentage of parental parasites binding to ICAM-1 or CD36. Assays were performed twice independently and *bars* represent median and interquartile ranges of samples tested in triplicate

Isolates 3D7 SBP1KO (SBP1 ‘knock-out’) and CS2 SBP1KO have been previously generated and characterized [20, 21]; the transport of PfEMP1 is arrested at MCs or the parasitophorous vacuole, knob formation occurs normally, but IE adhesion by SBP1KO was substantially reduced [20, 21]. In this study, we generated an additional parasite isolate, E8B SBP1KO. E8B parental [27], repeatedly selected for adhesion to immobilized intercellular adhesion molecule-1 (ICAM-1), was transfected with the *pHHT*-*TK*-*ΔSBP1* plasmid and human-dihydrofolate reductase (DHFR) cassette [20], which encodes resistance to WR99210, and then cycled for integration. Southern blot analyses of the *pfsbp1* locus in E8B showed the predicted band sizes of the parental and disrupted loci containing the WR99210 resistance gene *hDHFR* inserted by double crossover recombination (Fig. 1a). Western blot analyses of IE membrane extracts from pigmented trophozoites confirmed the loss of SBP1 in E8B SBP1KO compared to E8B parental which had a band at approximately 50 kDa (Fig. 1b). Northern blot analysis showed the active transcription of *var* genes in E8B SBP1KO that were of similar size to E8B parental (Fig. 1c), which suggests that there has not been a switch in *var* gene expression, consistent with previous findings with SBP1KO parasites [20]. Pigmented trophozoites of E8B SBP1KO had markedly reduced adhesion, with almost no binding to immobilized ICAM-1 (Fig. 1d) and CD36 (Fig. 1e), consistent with the loss of PfEMP1 surface expression. It was shown in previous studies, with repeated failure to select SBP1KO parasites for cytoadhesion, that this phenotype is not due to *var* gene switching [20].

**Fig. 2:**
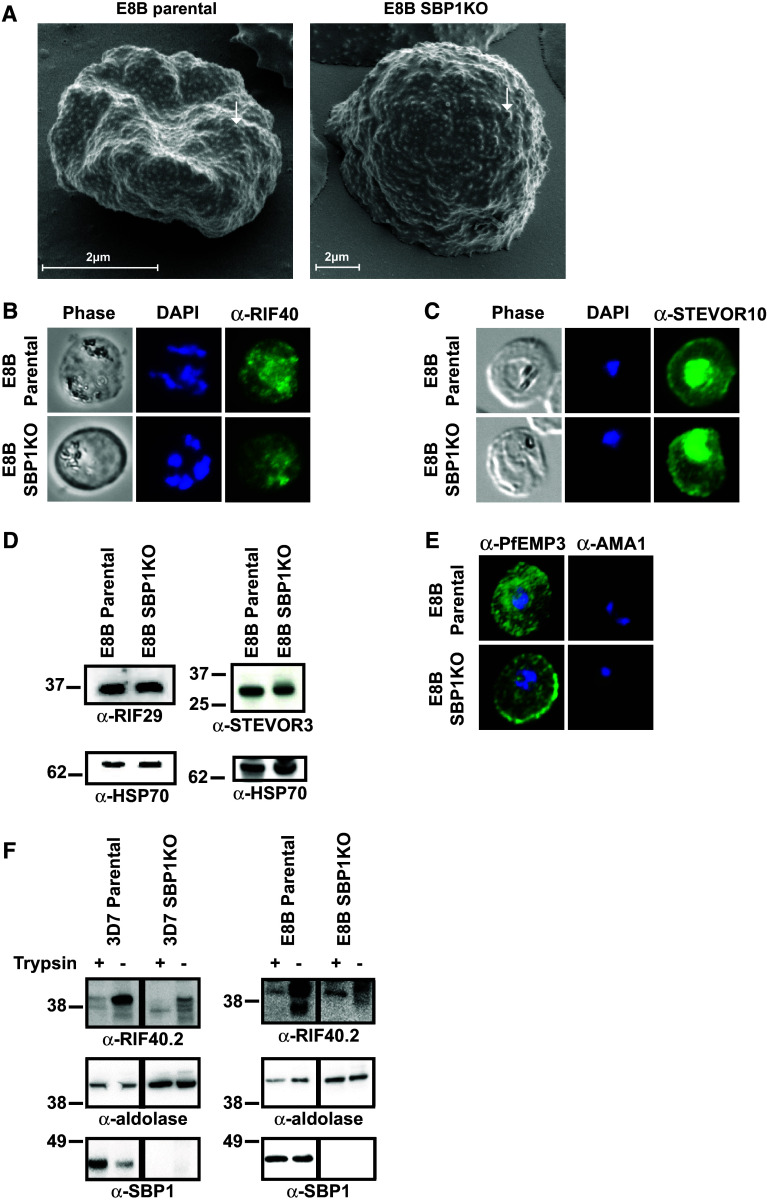
Ultrastructural features of the IE membrane and expression of IE membrane proteins by E8B parasites. **a** Scanning electron microscopy confirmed the expression of surface knob protrusions (*arrows*) with IEs from E8B SBP1KO similar to that of E8B parental. Representative images are shown and *scale* *bar* represents 2 μm in all images. Labeling of RIFIN (**b**) and STEVOR (**c**) proteins expressed by pigmented trophozoite-IEs were visualized by immunofluorescence microscopy using specific α-RIF40 and α-STEVOR10 antibodies (*green*). Despite the lack of PfEMP1 expression, RIFIN and STEVOR were detected in E8B SBP1KO (*lower* *panel*), similar to E8B parental (*upper* *panel*). The pattern of staining was consistent with the reported labeling of RIFIN and STEVOR in the IE membrane. **d** Western blot analyses of pigmented trophozoite-IEs membrane extracts probed with α-RIF29 (*left*) and α-STEVOR3 (*right*) antibodies, which detected RIFIN and STEVOR in both E8B parental and E8B SBP1KO parasites. Equal loading of parasite material was confirmed with α-HSP70 antibodies (*bottom* *panel*). **e** Pigmented trophozoite-IEs of E8B parental and E8B SBP1KO were probed with α-PfEMP3 antibodies (first column) as a positive control and α-AMA1 antibodies (second column) as a negative control (antibody staining in green, DAPI staining in *blue*). As expected, the pattern of staining was consistent with the labeling of PfEMP3 in the IE membrane and there was no apparent labeling by anti-AMA1. In all immunofluorescence assays, cells were fixed with a mixture of acetone (90 %) and methanol (10 %) and DAPI was used to stain nuclear DNA (*blue*). All images were taken at equal exposure for both *parasite* *lines* and representative images are shown. **f** Western blot analyses of protein extracts from purified pigmented trophozoite-IEs (parental and SBP1KO) that were treated with trypsin (+) or control (−). Blots were probed with α-RIF40.2, α-aldolase and α-SBP1 for 3D7 (*left*) and E8B (*right*) parasites. There was a reduction in the labeling of RIFINs after trypsin treatment of live IEs. Equal loading of parasite material was confirmed with α-aldolase. α-SBP1 confirmed the absence of SBP1 protein in the SBP1KO parasites and showed that the erythrocyte plasma membrane remained intact during trypsin treatment

**Fig. 3:**
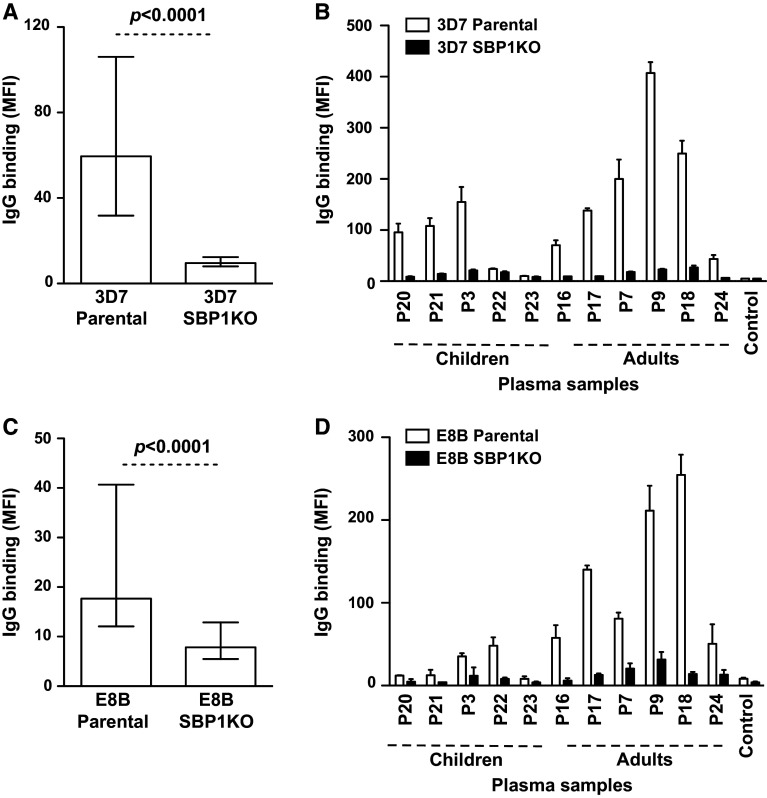
Antibodies among children and adults from PNG to surface antigens expressed by *P. falciparum*-IEs. IgG binding to the surface of erythrocytes infected with 3D7 SBP1KO (**a**), E8B SBP1KO (**c**) and was markedly reduced compared to 3D7 parental and E8B parental parasites, respectively. Assays were performed twice independently; *bars* represent median and interquartile ranges of samples tested in duplicate (*n* = 87 for 3D7, *n* = 91 for E8B); *p* value was calculated using a paired Wilcoxon signed rank test; IgG binding levels are expressed as geometric mean fluorescence intensity (MFI) for all graphs. A representative selection of serum samples from PNG tested for antibodies to 3D7 SBP1KO (**b**) and E8B SBP1KO (**d**). Samples were from malaria-exposed children and adults (P3-P25) (**b** and **d**) and non-exposed Melbourne residents (Control). IgG binding to 3D7 SBP1KO and E8B SBP1KO was substantially reduced in most individuals, with adults having a higher proportion of antibody response. There was minimal background reactivity observed among sera from Melbourne residents. Assays were performed twice independently; *bars* represent mean and range of samples tested in duplicate

To further characterize the different SBP1KO parasites, it was important to investigate whether the deletion of *pfsbp1* had affected the trafficking and expression of other IE membrane proteins. 3D7 SBP1KO and CS2 SBP1KO were previously reported to retain the typical expression of knobs similar to parental parasites, suggesting that KAHRP expression was unaffected [20, 21]. Here, scanning electron microscopy of pigmented trophozoite-IEs confirmed the expression of knobs by E8B SBP1KO similar to those of E8B parental (Fig. 2a). Additionally, RIFIN and STEVOR proteins localized to the IE membrane remain expressed by both E8B SBP1KO (Fig. 2) and 3D7 SBP1KO (Supplemental Fig S1), similar to their respective parental parasites. Immunofluorescence microscopy with anti-RIF40 antibodies labeled the IE membrane of E8B (Fig. 2b) and 3D7 mature trophozoites (Supplemental Fig S1A). Anti-STEVOR10 antibodies also labeled the IE membrane of mature trophozoites from E8B parental and SBP1KO (Fig. 2c) and 3D7 parental and SBP1KO isolates (Supplemental Fig S1B) by immunofluorescence microscopy. There was no labeling of uninfected erythrocytes by anti-RIF40 or anti-STEVOR10 antibodies and cells were fixed prior to antibody incubations. Furthermore, anti-RIF29 and anti-STEVOR3 antibodies labeled proteins of expected molecular size (~30–40 kDa) in Western blots of IE membrane extracts from E8B (Fig. 2d) and 3D7 mature trophozoites (Supplemental Fig S1C). Immunofluorescence microscopy also confirmed that another IE membrane protein, PfEMP3, remained expressed by E8B SBP1KO (Fig. 2e) and 3D7 SBP1KO (Supplemental Fig S1D), similar to parental parasites. We obtained further evidence that RIFINs remain exposed on the IE surface of the SBP1KO parasites. We examined degradation of RIFINs following treatment of intact IEs with high concentrations of trypsin (1 mg/ml for 30 min), which has been reported to cleave RIFINs [28–30]. Western blotting of IE extracts from pigmented trophozoites with anti-RIF40.2 antibodies demonstrated that trypsin treatment of intact 3D7 parental and 3D7 SBP1KO IEs led to a reduction in the labeling of RIFINs, consistent with the cleavage of RIFINs on the IE surface by trypsin (Fig. 2f). Similarly, trypsin treatment of intact E8B parental and E8B SBP1KO led to a reduction of RIFINs labeled on western blots, suggesting cleavage of surface-exposed RIFIN proteins (Fig. 2f). These findings suggest that the trafficking and expression of several IE membrane proteins other than PfEMP1 occurred normally in the SBP1KO parasites.

### Antibodies to the IE surface predominantly target antigens dependent on SBP1 for trafficking

To understand the significance of SBP1 in the trafficking of IE surface antigens that are targeted by naturally acquired antibodies, samples from malaria-exposed individuals were measured by an established flow cytometry-based assay [15] against parental and SBP1KO parasites. The levels of acquired antibodies targeting native antigens expressed on the IE surface were quantified.

## Papua New Guinea (PNG)

We tested a selection of serum samples (*n* = 87) from malaria-exposed adults (*n* = 46) and children (*n* = 41) residing in Madang, PNG for antibodies to the surface of erythrocytes infected with 3D7 parental and 3D7 SBP1KO parasites. The overall IgG binding to 3D7 SBP1KO was substantially reduced compared to 3D7 parental (Fig. 3a; *p* < 0.0001). Sera from most individuals showed a marked reduction in IgG binding to 3D7 SBP1KO compared to 3D7 parental (Fig. 3b). Of those samples that were considered positive for IgG binding (defined as antibody levels greater than mean + 3SD of negative controls) to 3D7 parental (81/87; 93.1 %), there was an 85.6 % reduction in median IgG binding to 3D7 SBP1KO (*p* = 0.01), suggesting that most antibodies are directed at SBP1-dependent antigens expressed on the IE surface (reflected by the level of IgG binding to 3D7 parental minus 3D7 SBP1KO). To further understand the role of SBP1 in the transport of IE surface antigens, we used the genetically different parasite isolate, E8B. A selection of plasma samples (*n* = 91) from the same PNG cohort of adults (*n* = 50) and children (*n* = 41) were tested for antibodies to the surface of erythrocytes infected with E8B parental and E8B SBP1KO parasites. The overall IgG binding to E8B SBP1KO was greatly reduced compared to E8B parental (Fig. 3c; *p* < 0.0001). Most plasma samples showed a substantial decrease in IgG binding to E8B SBP1KO (Fig. 3d). Of those samples that were considered positive for IgG binding to E8B parental (72/91; 79.1 %), there was a 61.3 % reduction in median antibody reactivity to E8B SBP1KO (*p* = 0.001), further indicating that the majority of the antibody response in this cohort was directed towards IE surface antigens that are dependent on SBP1 for trafficking.

Antibody levels to 3D7 parental in adults and children from PNG were largely similar (Supplemental Fig S2A; *p* = 0.15), suggesting that antibodies to IE surface antigens of 3D7 were acquired at a relatively young age in this population. IgG binding to 3D7 SBP1KO was markedly reduced compared to 3D7 parental for both adults (*p* < 0.0001) and children (*p* < 0.0001) (Supplemental Fig S2B), indicating that antibodies targeting SBP1-dependent antigens on the IE surface are highly prevalent in both groups. Adults had markedly higher IgG levels than children to E8B parasites (Supplemental Fig S2C; *p* = 0.0004). Similar to 3D7, IgG binding to E8B SBP1KO was substantially reduced compared to E8B parental for both adults (*p* < 0.0001) and children (*p* < 0.0001) (Supplemental Fig S2D). These results further indicate that naturally acquired human antibodies to the IE surface are primarily directed towards SBP1-dependent antigens, among children and adults.

## Kenya

**Fig. 4:**
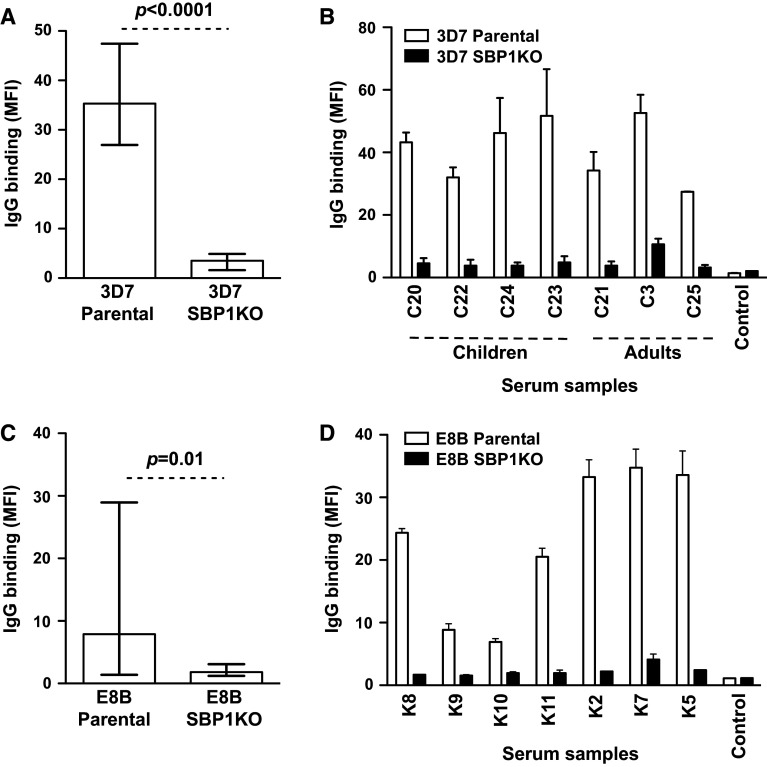
Antibodies among Kenyan individuals to surface antigens expressed by *P. falciparum*-IEs. IgG binding to the surface of erythrocytes infected with 3D7 SBP1KO (**a**) and E8B SBP1KO (**c**) was markedly reduced compared to 3D7 parental and E8B parental. Assays were performed twice independently; *bars* represent median and interquartile ranges of samples tested in duplicate (*n* = 26 for 3D7, *n* = 15 for E8B); *p* value was calculated using a paired Wilcoxon signed rank test; IgG binding levels are expressed as geometric mean fluorescence intensity (MFI) for all graphs. A representative selection of serum samples tested for antibodies to 3D7 SBP1KO (**b**) and E8B SBP1KO (**d**). Samples were from malaria-exposed individuals residing in Kenya (C3–C25; K2–K11) and non-exposed Melbourne residents (control). IgG binding to 3D7 SBP1KO and E8B SBP1KO was substantially reduced in most individuals. There was minimal background reactivity observed among sera from Melbourne residents. Assays were performed twice independently; *bars* represent mean and range of samples tested in duplicate

To expand these findings in a geographically different population, serum samples (*n* = 26) from a selection of adults (*n* = 5) and children (*n* = 21) residing in Chonyi, Kenya were tested for antibodies to the surface of erythrocytes infected with 3D7 parental and 3D7 SBP1KO parasites. The overall IgG binding to 3D7 SBP1KO was substantially reduced compared to 3D7 parental (Fig. 4a; *p* < 0.0001). Most individuals showed a marked reduction in IgG binding to 3D7 SBP1KO (Fig. 4b). All 26 samples were classified as positive for IgG binding to 3D7 parental and there was a 90 % reduction in median antibody reactivity to 3D7 SBP1KO (*p* = 0.0002). Serum samples (*n* = 15) from adults residing in Kilifi, Kenya were also tested for antibodies to the surface of erythrocytes infected with E8B parental and E8B SBP1KO. The overall IgG reactivity to E8B SBP1KO was markedly reduced compared to E8B parental (Fig. 4c) (*p* = 0.01). Sera from most individuals showed a substantial reduction in IgG binding to E8B SBP1KO (Fig. 4d). All 15 samples were classified as positive for IgG binding to E8B parental and there was a 81.9 % reduction in median antibody reactivity to E8B SBP1KO (*p* = 0.15), suggesting that SBP1 is crucial for the trafficking of major antibody targets.

### Antibodies to pregnancy-associated surface antigens predominantly target SBP1-dependent antigens

**Fig. 5:**
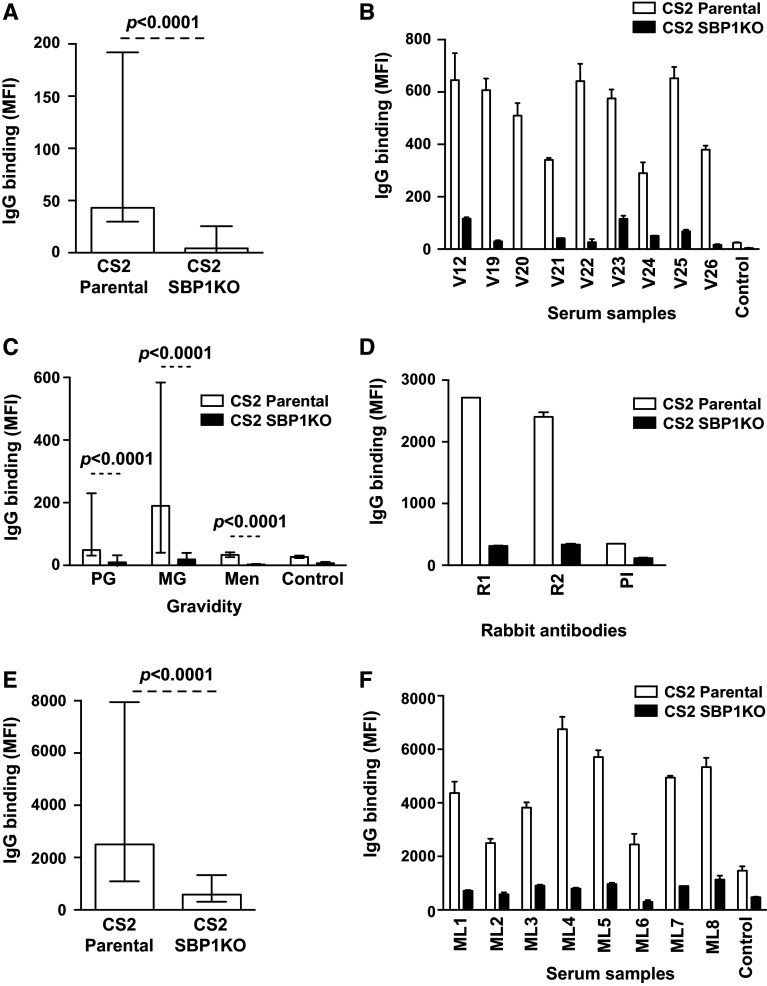
Antibodies among pregnant women and men from PNG and Malawi to surface antigens expressed by *P. falciparum*-IEs. **a** Among PNG donors, IgG binding to the surface of erythrocytes infected with CS2 SBP1KO parasites was markedly reduced compared to CS2 parental. Assays were performed twice independently; *bars* represent median and interquartile ranges of samples tested in duplicate (*n* = 94); *p* value was calculated using a paired Wilcoxon signed rank test; IgG binding levels are expressed as geometric mean fluorescence intensity (MFI) for all graphs. **b** A representative selection of samples from PNG pregnant women tested for antibodies to CS2 parental and CS2 SBP1KO. IgG binding to CS2 SBP1KO was substantially reduced in most individuals. There was minimal background reactivity observed among sera from non-exposed Melbourne residents (Control). Assays were performed twice independently; *p* value was calculated using a paired Wilcoxon signed rank test; *bars* represent mean and range of samples tested in duplicate. **c**. Serum samples from pregnant women in PNG tested for antibodies to CS2 parental and CS2 SBP1KO parasites are grouped by gravidity. Samples were from malaria-exposed primigravid (PG; *n* = 33) and multigravid (MG; *n* = 30) women and men (*n* = 31) in PNG. IgG binding to CS2 SBP1KO parasites was substantially reduced compared to CS2 parental in PG women, MG women and men. There was minimal background reactivity observed among sera from non-exposed Melbourne residents (Control). Assays were performed twice independently; *p* value was calculated using a paired Wilcoxon signed rank test; *bars* represent median and interquartile ranges of samples tested in duplicate. **d** Rabbit antibodies (R1–R2) raised against the DBL3 domain of VAR2CSA were tested for antibody recognition to CS2 parental and CS2 SBP1KO. All samples showed a marked reduction in IgG binding to CS2 SBP1KO. PI represents pre-immune serum from these rabbits. Assays were performed twice independently; *bars* represent mean and range of samples tested in duplicate. **e** Among pregnant women from Malawi, IgG binding to the surface of erythrocytes infected with CS2 SBP1KO parasites was markedly reduced compared to CS2 parental. Assay was performed once; *bars* represent median and interquartile ranges of samples tested in duplicate (*n* = 81); *p* value was calculated using a paired Wilcoxon signed rank test; IgG binding levels are expressed as geometric mean fluorescence intensity (MFI) for all graphs. **f** A representative selection of samples tested for antibodies to CS2 parental and CS2 SBP1KO. Samples were from malaria-exposed pregnant women from Malawi (ML1–ML8) and non-exposed Melbourne residents (Control). IgG binding to CS2 SBP1KO was substantially reduced in most individuals. There was minimal background reactivity observed among sera from non-exposed Melbourne residents (Control). Assay was performed once; *p* value was calculated using a paired Wilcoxon signed rank test; *bars* represent mean and range of samples tested in duplicate

Pregnant women are an additional group at high risk of malaria and are infected with specific *P. falciparum* variants that evade existing immunity and sequester in the placenta [31, 32]. Serum samples (*n* = 94) from a cohort of pregnant women residing in PNG were tested for antibodies to the IE surface of the placental-binding isolate CS2 parental and CS2 SBP1KO. This isolate expresses VAR2CSA, a PfEMP1 variant that mediates adhesion to CSA in the placenta [33]. The overall IgG binding to CS2 SBP1KO was substantially reduced (90 %) compared to CS2 parental (Fig. 5a; *p* < 0.0001). Most individuals showed a marked reduction in IgG binding to CS2 SBP1KO (Fig. 5b). When classified according to gravidity, the majority of samples tested from primigravid (PG; *n* = 33) and multigravid (MG; *n* = 30) women showed a marked decrease in antibody response to CS2 SBP1KO compared to CS2 parental (Fig. 5c); 19/33 samples (57.6 %) from PG women were positive for IgG binding to CS2 parental and 12/33 samples (36.4 %) were positive for IgG binding to CS2 SBP1KO (*p* = 0.09); 23/30 samples (76.7 %) and 15/30 samples (50 %) from MG women were positive for IgG binding to CS2 parental and CS2 SBP1KO, respectively (*p* = 0.05). Of those samples that were considered positive for IgG binding to CS2 parental, IgG binding to CS2 SBP1KO was reduced by 83.1 % in PG women and 91.2 % in MG women. As expected, the level of antibodies was substantially higher in MG women than PG women (Fig. 5c), consistent with previous reports [31, 34]. The level and prevalence of antibodies to CS2 parasites were substantially lower in men compared to pregnant women (Fig. 5c); 9/31 samples (29 %) from men were classified as positive for IgG binding to CS2 parental whereas none of the samples were classified as positive for IgG binding to CS2 SBP1KO (*p* = 0.001).

We confirmed these findings with serum samples from a cohort of pregnant women (*n* = 71) and men (*n* = 10) residing in Malawi. Again, overall IgG binding to CS2 SBP1KO was substantially reduced (76.7 %) compared to CS2 parental (Fig. 5e; *p* < 0.0001). Most individuals showed a marked reduction in IgG binding to CS2 SBP1KO (Fig. 5f). When classified according to gravidity, the majority of samples tested from primigravid (PG; *n* = 35) and multigravid (MG; *n* = 36) women showed a marked decrease in antibody response to CS2 SBP1KO compared to CS2 parental (Supplemental Fig S3A); 22/35 samples (62.9 %) from PG women were positive to CS2 parental and 20/35 samples (57.1 %) were positive to CS2 SBP1KO (*p* = 0.73); 22/36 samples (61.1 %) from MG women were positive for IgG to CS2 parental while 6/36 samples (16.7 %) were positive for CS2 SBP1KO (*p* = 0.001). The overall IgG binding to CS2 SBP1KO in samples from men residing in the same region was markedly reduced compared to CS2 parental (Supplemental Fig S3A; *p* = 0.002). The level and prevalence of antibodies to CS2 parasites were substantially lower in men compared to pregnant women (Supplemental Fig S3A).

To confirm the loss of VAR2CSA-PfEMP1 expression in CS2 SBP1KO, we tested rabbit antibodies raised against the DBL3 domain of VAR2CSA expressed by CS2 [35] for recognition of the IE surface of CS2 parental and CS2 SBP1KO parasites. There was markedly lower IgG binding to CS2 SBP1KO (Fig. 5d), suggesting the absence of PfEMP1 on the IE surface of these parasites and supporting the significance of SBP1 in the trafficking of PfEMP1.

### Antibodies that mediate opsonic phagocytosis of IEs predominantly target SBP1-dependent antigens on the IE surface

**Fig. 6:**
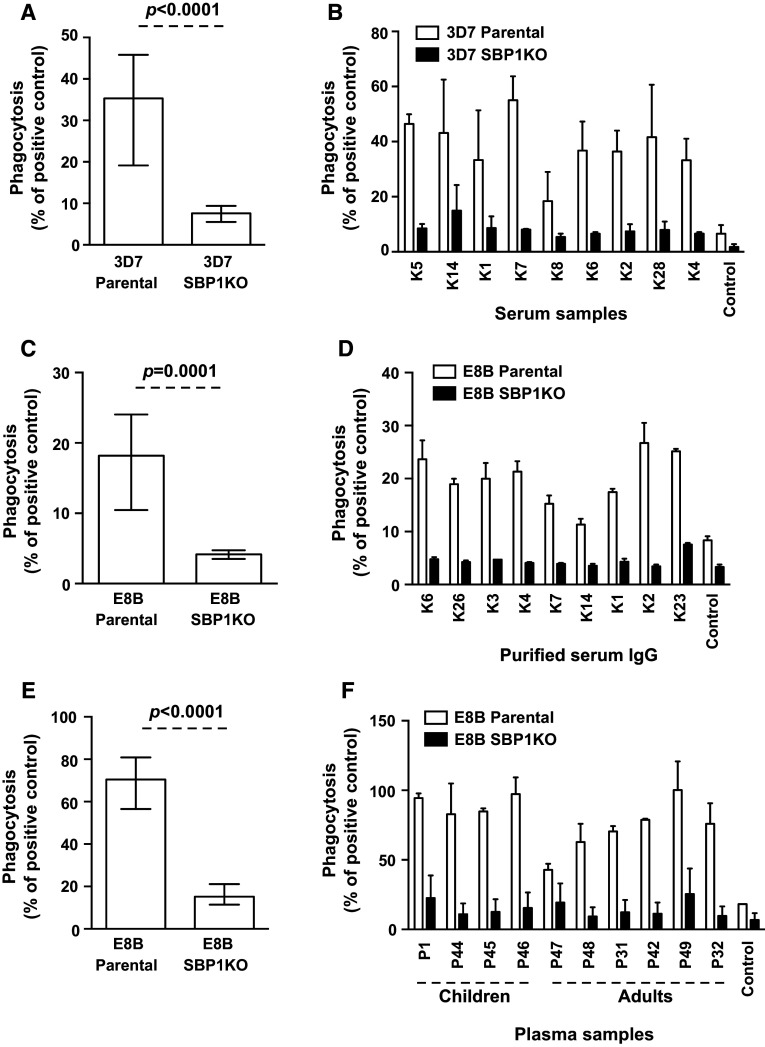
Opsonic phagocytosis of IEs by undifferentiated Thp-1 monocytes. Opsonic phagocytosis activity of serum antibodies from Kenya and PNG was markedly reduced with 3D7 SBP1KO (**a**) and E8B SBP1KO (**c**, **e**) compared to 3D7 parental and E8B parental. Assays were performed twice independently; *bars* represent median and interquartile ranges [for Kenyan samples, *n* = 25 for 3D7 (**a**), *n* = 9 for E8B (**b**); for PNG samples, *n* = 25 for E8B (**c**)]; *p* value was calculated using a paired Wilcoxon signed rank test. For all graphs, the level of opsonic phagocytosis is expressed as a percentage of positive control (rabbit antibody raised against human erythrocytes). A representative selection of serum samples from Kenya tested for opsonic phagocytosis activity to 3D7 SBP1KO (**b**) and E8B SBP1KO (**d**) and from PNG tested for opsonic phagocytosis activity to E8B SBP1KO (**f**). Assays were performed twice independently for 3D7 and once for E8B; *bars* represent mean and range with samples tested in duplicate

To examine the importance of SBP1 in the trafficking of IE surface antigens targeted by functional antibodies, serum samples from Kenyan adults were tested for their ability to opsonize pigmented trophozoite-IEs for phagocytic clearance by undifferentiated Thp-1 monocytes, an established model of opsonic phagocytosis [15, 36, 37]. IEs were incubated with serum samples for opsonisation by antibodies before co-incubation with THP-1 monocytes was performed to test for phagocytosis. The ability of serum antibodies (*n* = 25) to promote opsonic phagocytosis activity was substantially reduced (78.4 %) in 3D7 SBP1KO compared to 3D7 parental (Fig. 6a; *p* < 0.0001). We also tested serum IgG purified from these Kenyan adults (*n* = 9) for their opsonic phagocytosis activity with E8B parental and E8B SBP1KO. The level of opsonic phagocytosis was markedly reduced by 77.1 % in E8B SBP1KO compared to E8B parental (Fig. 6c; *p* = 0.0001). All samples showed a reduction in opsonic phagocytosis activity to 3D7 SBP1KO (Fig. 6b) and E8B SBP1KO (Fig. 6d) compared to their respective parental parasites. Furthermore, we tested a selection of plasma samples (*n* = 28) from adults and children residing in Madang, PNG for their opsonic phagocytosis activity. Similarly, the ability of serum antibodies to opsonize IEs for phagocytosis was substantially reduced (60.8 %) in E8B SBP1KO compared to E8B parental (Fig. 6e; *p* < 0.0001) and all samples showed a reduction in opsonic phagocytosis activity to E8B SBP1KO (Fig. 6f) compared to E8B parental. Together, our results suggest that antibodies target SBP1-dependent antigens on the IE surface function to opsonise IEs for phagocytosis.

**Fig. 7:**
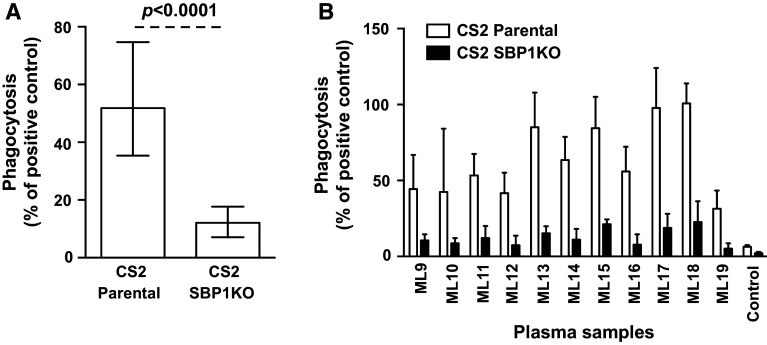
Opsonic phagocytosis of IEs by undifferentiated Thp-1 monocytes using sera from pregnant women in Malawi. Opsonic phagocytosis activity was markedly reduced in CS2 SBP1KO (**a**) compared to CS2 parental. Assays were performed twice independently; *bars* represent median and interquartile ranges (*n* = 36); *p* value was calculated using a paired Wilcoxon signed rank test. For all graphs, the level of opsonic phagocytosis is expressed as a percentage of positive control (rabbit antibody raised against human erythrocytes). A representative selection of serum samples tested for opsonic phagocytosis activity to CS2 SBP1KO (**b**). Samples were from malaria-exposed pregnant women residing in Malawi (ML9–19) and non-exposed Melbourne residents (Control). Assays were performed twice independently; *bars* represent mean and range with samples tested in duplicate

We tested serum samples from pregnant women (*n* = 36) residing in Malawi for their opsonic phagocytosis activity to CS2 parental and CS2 SBP1KO parasites. The ability of serum antibodies to promote opsonic phagocytosis activity was substantially reduced (76.4 %) in CS2 SBP1KO compared to CS2 parental (Fig. 7a; *p* < 0.0001). All samples showed a reduction in opsonic phagocytosis activity to CS2 SBP1KO (Fig. 7b) compared to their respective parental parasites, suggesting the functional importance of antibodies to SBP1-dependent antigens.

### PfEMP1 is the major target of antibodies to the IE surface

**Fig. 8:**
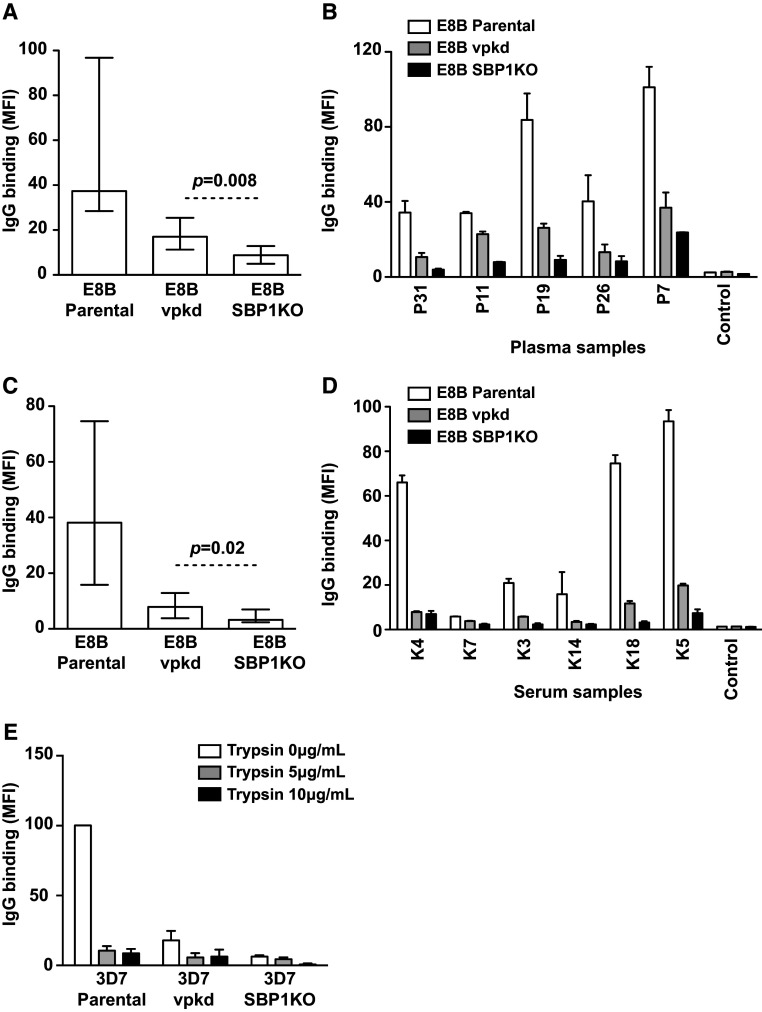
PfEMP1 is the major target of human antibodies. The overall IgG binding to E8B SBP1KO parasites was markedly reduced compared to E8B parental parasites and further reduced compared to E8Bvpkd, among samples from PNG (**a**) and Kenya (**c**). Assays were performed twice independently; *bars* represent median and interquartile ranges (*n* = 12 for PNG, *n* = 7 for Kenya); *p* value was calculated using a paired Wilcoxon signed rank test; IgG binding levels are expressed as geometric mean fluorescence intensity (MFI) for all graphs. A representative selection of samples tested in parallel for antibodies to E8B parental, E8Bvpkd and E8B SBP1KO. Samples were from malaria-exposed PNG adults (**b**; P7–P31), Kenyan adults (**d**; K3–K18) and non-exposed Melbourne residents (Control). There was minimal background reactivity observed among sera from Melbourne residents. Assays were performed twice independently; *bars* represent mean and range of samples tested in duplicate. **e** IgG binding to the IE surface of 3D7 parental and 3D7vpkd was trypsin sensitive compared to 3D7 SBP1KO. IEs were incubated with trypsin at 5 or 10 ug/ml, or without trypsin, and tested for IgG reactivity among samples form Kenyan adults. IgG binding levels are expressed as a percentage of their respective 3D7 parental reactivity without trypsin; *bars* represent mean and SEM of samples tested in duplicate (*n* = 5) after subtraction with IgG levels from negative Melbourne controls

To investigate the identity of SBP1-dependent antigens recognized by human antibodies we used *P. falciparum* that was genetically engineered to suppress PfEMP1 expression (*var* promoter knock-down, vpkd). These parasites, which have been previously described and characterized in detail, have inhibited PfEMP1 surface expression through the suppression of endogenous *var* genes [15, 38]. Antibody levels to the surface of pigmented trophozoite-IEs were compared between parental, vpkd and SBP1KO parasites. In a selection of PNG individuals (*n* = 12), the overall IgG reactivity to E8Bvpkd and E8B SBP1KO was substantially lower than parental E8B. Reactivity to E8B SBP1KO was lower again than to E8Bvpkd (Fig. 8a; *p* = 0.008). Compared to E8B parental, IgG binding was reduced by 54.5 % to E8Bvpkd and by 76.5 % to E8B SBP1KO. The majority of the samples showed a marked reduction in IgG binding to E8B SBP1KO compared to E8Bvpkd and E8B parental (Fig. 8b). Similar results were seen using adult sera from Kilifi, Kenya (*n* = 7). (Figure 8c, d; *p* = 0.02). Compared to E8B parental, IgG binding was reduced by 79.3 % to E8Bvpkd and by 91.6 % to E8B SBP1KO. Similar results were also seen using 3D7 parental, vpkd and SBP1KO parasites (Supplemental Fig S4). For PNG serum (*n* = 81) IgG binding was reduced by 79.5 % to 3D7vpkd and by 89 % to 3D7 SBP1KO compared to parental 3D7 (Supplemental Fig S4A, S4B). For samples from Kenya, IgG binding was reduced by 76.7 % to 3D7vpkd and by 90 % to 3D7 SBP1KO compared to 3D7 parental (Supplemental Fig S4C, S4D). This suggests that most of the SBP1-dependent antibody targets are PfEMP1.

To understand the nature of residual antibody reactivity to vpkd IEs, compared to SBP1KO IEs, we evaluated the effect of trypsin treatment of IEs on antibody binding (Fig. 8e). The majority of IgG binding to 3D7 parental surface antigens was highly trypsin sensitive, suggesting that antibodies are targeting PfEMP1 on the IE surface, since PfEMP1 is known to be trypsin sensitive [4]. The residual IgG binding observed with 3D7vpkd parasites was also trypsin sensitive, consistent with PfEMP1 being the target of these antibodies. Treatment of 3D7vpkd with trypsin reduced antibody reactivity to levels that were comparable to 3D7 SBP1KO, suggesting that antibody reactivity to 3D7vpkd may represent residual PfEMP1 expression.

## Discussion

Our findings indicate that, remarkably, a single parasite protein, SBP1, is required for the transport of IE surface antigens that are the major targets of human antibodies against *P. falciparum* infections. SBP1 is clearly a crucial component of the trafficking pathway for surface antigens and may represent an important difference between *P. falciparum* and other human malaria species. Comparing the level of IgG binding to the IE surface between parental and SBP1KO allowed the quantification of antibodies targeting SBP1-dependent antigens and defined a key property of major surface antigens and epitopes. The overall IgG binding to SBP1KO IEs was dramatically reduced compared to parental parasites, indicating that the great majority of the naturally acquired antibodies to the IE surface targeted SBP1-dependent antigens. The decrease in antibody reactivity to SBP1KO parasites was observed with serum antibodies from children and adults from PNG and Kenya, as well as serum antibodies from pregnant women residing in PNG and Malawi. Furthermore, the level of functional antibodies measured through opsonic phagocytosis activity was greatly reduced in the SBP1KO parasites. Comparisons between antibody reactivity to SBP1KO and PfEMP1 knock-down (vpkd) parasites supported the conclusion that PfEMP1 is the dominant target of human antibodies.

*Plasmodium falciparum* malaria affects children, adults and pregnant women, and has a high burden of disease in many parts of Africa, Asia, South America and the Pacific. *Plasmodium falciparum* exposure and the development of naturally acquired immunity can vary considerably between risk groups and geographical regions. Therefore, to ensure generalizability of findings we included samples from all three risk groups (children, adults, pregnant women) that represented different populations from Africa (Kenya and Malawi) and other regions (PNG). Additionally, we used three different parasite isolates, with different genetic or phenotypic backgrounds. Prior studies have shown that parasite isolates such as 3D7 and IT4 lines (E8B) are commonly recognized by serum from malaria-exposed individuals across different geographic regions [27, 39, 40]. Strikingly, the same pattern of substantially reduced antibody binding to SBP1KO parasites was observed in all risk groups and populations with each of our three *P. falciparum* isolates. These repeated observations provide strong evidence for SBP1-dependent IE surface antigens being predominant antibody targets with minimal targeting of non-SBP1-dependent antigens by human immune responses.

The functional mechanisms of antibodies to IE surface antigens that protect against malaria are not fully understood. Antibodies are believed to contribute to protection against symptomatic disease by opsonising IEs for clearance by monocytes or macrophages [25, 26, 41]. Undifferentiated THP-1 monocytes were used in this study because they specifically measure Fc-mediated opsonic phagocytosis and have very little non-opsonic phagocytosis [36]. Antibody-mediated opsonic phagocytosis was greatly reduced in 3D7 SBP1KO, E8B SBP1KO and CS2 SBP1KO IEs compared to their respective parental parasites, providing key evidence that antibodies to SBP1-dependent epitopes on the IE surface function in the opsonic phagocytosis of IEs. It was interesting that some measurable opsonic phagocytosis activity was still detected in the SBP1KO parasites. Although it was low, it may still contribute to immunity and could potentially be accounted for by antibodies to other IE surface antigens such as RIFIN and STEVOR, which are still expressed by the SBP1KO parasites. An additional important mechanism that is thought to contribute to immunity is antibody inhibition of IE adhesion or rosetting (reviewed in [5]). For example, antibodies from pregnant women sera inhibited the binding of IEs to the placental receptor CSA [34, 42], which have been associated with reduced malaria in pregnancy and placental infection [42, 43]. Furthermore, antibodies from children with mild malaria disrupted rosette formation whereas those with severe malaria did not, suggesting that acquired antibodies mediate protection through inhibition of rosette formation [44, 45]. In our study, and previous studies, we show that disruption of SBP1 leads to loss of cytoadhesion ability and antibody recognition, suggesting that antibodies that inhibit adhesion are targeting SBP1-dependent antigens, particularly PfEMP1. IE surface antigens also have other important immune interactions, such as cellular immunity. Prior studies have reported that PfEMP1 may impact on responses by a range of cell types, including dendritic cells, T cells, and innate immune cells [46–50]. Future studies using SBP1KO parasites to better understand these responses may be valuable for understanding the role of PfEMP1 and other surface antigens in cell-mediated immunity.

Antibodies directed to the IE surface of *P. falciparum* isolates that are prevalent in pregnancy malaria also represent important components of protective immunity [42]. These antibodies are thought to confer protection by inhibiting parasite sequestration in the placenta [31, 42, 51] and promoting the opsonic phagocytosis of IEs [52–54]. The PfEMP1 variant, VAR2CSA, is an important antibody target and vaccine candidate but other antigens have also been proposed [55]. Reflecting previous exposure to CSA-binding isolates that primarily expresses *var2csa* [56], multigravid (MG) women generally have a higher prevalence of antibodies to placental isolates compared to primigravid (PG) women or men [31, 34, 57] and the findings in this study are consistent with that. IgG binding to CS2 SBP1KO in both PG and MG women was markedly reduced compared to CS2 parental, demonstrating that surface antigens dependent on SBP1 are major targets of acquired antibodies, a substantial proportion of which is presumably directed at VAR2CSA. In our study, rabbit antibodies raised against VAR2CSA were used to confirm the loss of PfEMP1 surface expression in the CS2 SBP1KO parasites.

The importance of PfEMP1 as a target was demonstrated by comparing the levels of IgG binding between parental, vpkd and SBP1KO parasites. The majority of the loss of reactivity of antibodies to SBP1KO parasites could be explained by a loss of PfEMP1, supporting the conclusion that PfEMP1 is the major target of human antibodies to the IE surface. Interestingly, there was a small further reduction in antibody reactivity to SBP1KO compared to vpkd parasites. This was observed in E8B and 3D7 parasites, and similarly for both PNG and Kenyan populations. The residual antibody binding to vpkd compared to SBP1KO might reflect residual PfEMP1 expression in the vpkd parasites, whereas SBP1KO may have inhibited PfEMP1 surface display more efficiently. Reactivity to 3D7vpkd was largely trypsin sensitive, similar to the sensitivity pattern of 3D7 parental parasites, suggesting that the reactivity may be explained by residual PfEMP1 expression on vpkd parasites. PfEMP1 has been described as being highly sensitive to trypsin treatment [4] compared to other antigens such as RIFIN, that have been reported to be resistant to cleavage by low trypsin concentrations [28, 29].

The low antibody reactivity to SBP1KO parasites suggests that antibodies that may target other antigens that are trafficked to the IE surface independent of SBP1 expression are not a prominent feature of the naturally acquired immune response. Possible antigens include RIFIN, STEVOR or SURFIN families which have been previously identified on the IE surface [12, 13, 28–30, 58, 59]. We found that that the expression of other surface proteins, such as RIFIN and STEVOR, were similar in the parental and SBP1KO parasites, suggesting that these surface antigens are not dependent on SBP1. We also provided evidence that RIFINs were exposed on the IE surface, by showing that trypsin treatment of intact IEs led to degradation of RIFINs. Furthermore, exported proteins required for knob formation were still expressed by the SBP1KO parasites. There is a possibility that the expression levels of RIFINs and STEVORs may have changed in the SBP1KO parasites compared to parental and this may contribute to reduced antibody recognition. One previous study [58] reported that a lower proportion of IEs stained positive with STEVOR antibodies (raised against a STEVOR peptide) for 3 lab isolates compared to 4 recently adapted isolates. Currently we lack the tools to quantify the expression of these other antigens on the IE surface of parental and SBP1KO parasites, but there did not appear to be a major difference in expression between parental and KO parasites using western blots and immunofluorescence microscopy, although these methods are not highly quantitative. Rapidly generating SBP1KO clones with clinical isolates is currently not possible. It is unlikely that our findings of reduced antibody recognition of SBP1KO IEs can be explained by the switching of *var* genes and PfEMP1 variants expressed between parental and SBP1KO parasites as Northern blots showed bands of similar sizes in both parental and SBP1KO parasites, and the disruption of surface-exposed PfEMP1 has been confirmed in three independent parasite isolates. Furthermore, the immune serum used in our assays is not variant-specific and should detect a wide repertoire of PfEMP1 variants, should they be expressed by the SBP1KO parasites. In addition, previous studies have been unable to select the SBP1KO parasites for adhesion to immobilized receptors, thus suggesting that the parasites have not switched to another PfEMP1 variant [20, 21].

These findings have important translational implications for understanding and measuring immunity, and developing interventions against malaria. A strong knowledge of the targets of mechanisms of immunity is crucial for the development of effective vaccines, and our findings further highlight the significance of PfEMP1 as the major target of acquired human antibodies. While PfEMP1 is a known target of immunity, it is important to quantify its role relative to other antigens in order to prioritize antigens for vaccine development, and develop tools to quantify and monitor human immunity, which are currently lacking. There is significant interest in PfEMP1-based vaccines [60–62] and vaccines based on the VAR2CSA-PfEMP1 are progressing to clinical trials. We also demonstrate the value of SBP1KO parasites as a tool to measure and evaluate antibodies to surface antigens, particularly PfEMP1, in populations and potentially in vaccine trials. Unlike other tools, such as isolates with suppressed PfEMP1 expression [15, 38], *pfsbp1* gene-KO parasites are phenotypically stable in culture and can be readily applied in different assays and laboratories. Approaches to developing highly efficacious second generation vaccines include the potential use of attenuated whole parasites, including attenuated IEs [63, 64]. Therefore, it is important to define key virulence features and antibody targets. Moreover, our studies highlight a crucially important evolutionary difference between *P. falciparum* and *P. vivax*, which has major implications for understanding immunity and disease pathogenesis and the development of future vaccine and therapeutic strategies against these pathogens.

In conclusion, our study has provided major new evidence that the key targets of acquired human antibodies are highly dependent on SBP1 for trafficking to the IE surface. Moreover, finding that PfEMP1 is by far the major target of human antibodies to the IE surface supports efforts to develop vaccines based on this antigen. We also showed that antibodies to SBP1-dependent antigens, predominantly PfEMP1, play a key role in a mechanism of parasite clearance by opsonizing IEs for phagocytosis. These findings highlight an important role for SBP1 in the trafficking of IE surface antigens and define a key feature in the biology and virulence properties that may be different to other human malarias. These findings have major implications for malaria vaccine development, and for quantifying immunity in populations.

## Methods

### Study population and ethics statement

Serum samples (*n* = 100) were collected from a community-based cross-sectional study in the Madang Province, PNG [65]. This population experiences year-round malaria transmission with some seasonal variation [66]. Half of these samples were from children with a median age of 7 years (interquartile range, 6–9 years), while the other half were from adults with a median age of 28 years (interquartile range 24–35). For 9 subjects, there was insufficient serum available for inclusion in this study; therefore, a total of 91 subjects were included. Serum samples were also collected from a cross-sectional study of pregnant women in their second trimester (*n* = 63) who visited the antenatal clinic for routine care, and from men (*n* = 31), at the Yagaum Health Centre and Modilon Hospital in Madang Province, PNG as previously described [67, 68]. Of these, 33 samples from PGs and 30 samples from MG women were available for testing. In Malawi, samples were collected from pregnant women (*n* = 85) as described [69]. In Kenya, serum samples (*n* = 25) were also collected from anonymous adults at the Hospital Blood Donor Service in Kilifi, [15, 70] and from individuals participating in a longitudinal cohort study (*n* = 296) conducted in Chonyi, Kilifi district as described elsewhere [15, 70, 71]. Ethics approval was obtained from the Kenya Medical Research Institute Ethics Committee, the Medical Research Advisory Council PNG, the College of Medicine Research Ethics Committee in Malawi, the Walter and Eliza Hall Institute Human Research and Ethics Committee and the Alfred Hospital Human Research and Ethics Committee. Written informed consent was obtained from all study participants or their parents or legal guardians.

### *Plasmodium falciparum* culture and isolates

*Plasmodium falciparum* isolates were maintained in continuous culture and synchronized as previously described [15]. Isolate E8B SBP1KO was generated by transfecting E8B parental [27], derived from a clone of IT4, with the *pHHT*-*TK*-*ΔSBP1* plasmid and culturing in the presence of WR99210 (2.5 nM), using established methods [72]. Isolates 3D7 SBP1KO and CS2 SBP1KO were generated as previously described [20, 21]. Isolates 3D7vpkd and E8Bvpkd were generated as previously described [15, 38]. IEs were typically used in assays at the pigmented trophozoite developmental stage when they express the surface proteins PfEMP1, RIFIN, STEVOR, SURFIN (except ring stage parasites were used for transcript analysis by northern blotting).

### Measuring antibodies to the IE surface by flow cytometry

Measuring IgG binding to the IE surface of pigmented trophozoites was performed with an established flow cytometry-based assay as previously described [15]. IgG binding levels for each sample was expressed as the geometric mean fluorescence intensity (MFI; arbitrary units) for IEs, after subtracting the MFI of uninfected erythrocytes. Samples were tested at a 1/10 dilution and were designated antibody positive if the MFI was greater than the mean plus 3 standard deviation of reactivity seen with sera from non-malaria exposed Australian residents. Cryopreserved pigmented trophozoite IEs used in antibody assays have been previously validated (Beeson et al. unpublished) [15, 73]. Cryopreservation of IEs was performed as described [15]. IEs were incubated with various concentrations of trypsin (0–10 μg/mL; TPCK-treated, Worthington Biomedical) diluted in PBS for 15 min at 37 °C, as previously described [15].

### Western blots

Western blots of membrane proteins from pigmented trophozoite-IEs were performed using Triton-X 100-insoluble, SDS-soluble protein extracts of pigmented trophozoite-IEs as previously described [15]. Nitrocellulose membranes (Invitrogen) were probed with affinity-purified rat antibodies against recombinant RIF29 (1/2000) [74, 75], rat antibodies against RIF40.2 (1/2000) [30], affinity-purified mouse antibodies against recombinant STEVOR3 protein (1/2000) [76], rabbit anti-SBP1 (1/1000) [20], rabbit anti-HSP70 antibodies (1/500; provided by Paul Gilson) and rabbit anti-aldolase (1/5000; provided by Jake Baum). Magnet-purified IEs were incubated with trypsin at 1 mg/mL (TPCK-treated, Sigma–Aldrich) or PBS as a negative control for 30 min at 37 °C, and the reaction stopped with trypsin inhibitor (Sigma–Aldrich) before the cells were extracted with SDS loading buffer, as previously described [30].

### Northern blots

RNA from highly synchronous ring-staged parasite cultures at 10 h post-invasion was extracted using Trizol (Invitrogen) as previously described [15]. Northern blots were hybridized with a conserved *var* exon 2 sequence (PlasmoDB accession number PFIT_1219000) and washed at low stringency with 2X SSC and 0.1 % SDS at 55 °C [77].

### *Plasmodium falciparum* cytoadherence assays

Testing for adhesion to immobilized receptors was performed using gelatin-enriched pigmented trophozoite-IEs at 15–20 % parasitemia as previously described [15]. The numbers of bound cells were recorded as IEs per square millimeter and PBS was used as a negative control to record non-specific binding. Adhesion to each receptor was tested in triplicate at these concentrations: ICAM-1 10 μg/mL (Bender MedSystems) and CD36 20 μg/mL (rhCD36/Fc chimera, R&D systems).

### Immunofluorescence microscopy

Immunofluorescence microscopy of pigmented trophozoite-IEs were performed as previously described [15]. Slides were incubated with primary antibodies such as anti-A_RIF40_ (1/50; accession number AF483820) [75], anti-STEVOR10 (1/200) [76], anti-SBP1 (1/500) [20], anti-PfEMP3 (1/500; provided by Melanie Rug) and anti-AMA1 (apical membrane antigen 1; 1/500) [78], followed by the corresponding Alexa Fluor 488-conjugated IgG (1/500, Invitrogen). Images were obtained using a Plan-Apochromat (100 ×/1.40) oil immersion phase-contrast lens (Carl Zeiss) on an AxioVert 200 M microscope (Carl Zeiss) equipped with an AxioCam Mrm camera (Carl Zeiss).

### Opsonic phagocytosis assays

Measuring the level of opsonic phagocytosis activity using undifferentiated Thp-1 monocytes was conducted as previously described [15, 36]. The proportion of Thp-1 positive cells with phagocytosed pigmented trophozoite-IEs was counted by flow cytometry (FACSCalibur, BD Biosciences). The level of phagocytosis for each sample was expressed relative to the positive control (Abcam; rabbit antibody raised against human erythrocytes). Negative control samples from non-malaria exposed Australian residents were included in all assays. Purification of IgG was performed using Melon Gel IgG purification kit (Thermo Scientific) according to the manufacturer’s instructions and dialysed into PBS prior to performing antibody assays [79].

### Scanning electron microscopy

Square glass coverslips (22 mm) were prepared by smearing a 0.1 % solution of polyethyleneimine (PEI) and dried. Pigmented trophozoite-IE samples were incubated on PEI-coated glass coverslips for 30 min and then immersed in 2 % glutaraldehyde in PBS for 1 h. Coverslips were rinsed thrice in PBS for 10 min before being dehydrated in increasing concentrations of ethanol consisting of 10, 20, 40, 60, 80, and 100 % ethanol in water for 10 min each. The coverslips were then dried in a Balzers CPD 030 Critical Point Dryer (Balzers Pfeiffer, Balzers, Liechtenstein) and mounted onto 25 mm aluminum stubs with double-sided carbon tabs and then gold-coated in a Dynavac “Xenosput” magnetron sputter coater. Cells were imaged with the Philips XL30 field emission scanning electron microscope (Philips, Eindhoven, Netherlands) at a voltage of 2 kV.

### Statistical analyses

Non-parametric analytical methods were used to evaluate assay results from cohort studies, as the data obtained were not normally distributed. Differences in adhesion levels between parental and genetically modified parasites were assessed using an unpaired Mann–Whitney test. Differences in antibody levels between parental and genetically modified parasites were assessed using a paired Wilcoxon signed rank test or Kruskal–Wallis test (>2 groups). Statistical analyses were performed using Prism version 5 (GraphPad Software Inc) or Stata 12.0 (StataCorp).

### Electronic supplementary material

Below is the link to the electronic supplementary material.

**Supplemental Figure S1: Expression of IE membrane proteins by 3D7 parasites.** RIFIN (**A**) and STEVOR (**B**) proteins expressed by pigmented trophozoite-IEs of 3D7 parental and SBP1KO were visualized by immunofluorescence microscopy using specific α-RIF40 and α-STEVOR10 antibodies (green). RIFIN and STEVOR were detected in 3D7 SBP1KO (middle panel), similar to E8B parental (upper panel). The pattern of staining was consistent with the reported labeling of RIFIN and STEVOR in the IE membrane. The antibodies did not show any labeling of uninfected erythrocytes (uRBC) present in the culture (lower panel).**C**. Western blot analyses of pigmented trophozoite-IEs membrane extracts probed with α-RIF29 (left) and α-STEVOR3 (right) antibodies, which detected RIFIN at 40 kDa and STEVOR at 30 kDa in both 3D7 parental and 3D7 SBP1KO. Equal loading of parasite material was confirmed with anti-HSP70 antibodies (bottom panel). **D**. Pigmented trophozoite-IEs of 3D7 parental and 3D7 SBP1KO were probed with α-PfEMP3 antibodies (first column) as a positive control and α-AMA1 antibodies (second column) as a negative control (antibody staining in green, DAPI staining in blue). As expected, the pattern of staining was consistent with the labeling of PfEMP3 in the IE membrane and there was no apparent labeling of AMA1. In all immunofluorescence assays, cells were fixed with a mixture of acetone (90 %) and methanol (10 %) and DAPI was used to stain nuclear DNA (blue). All images were taken at equal exposure for both parasite lines and representative images are shown. (PDF 426 kb)

**Supplemental Figure S2:** **Comparing the antibody response between adults and children from PNG. A.** The level of IgG reactivity to the IE surface of 3D7 parental and 3D7 SBP1KO parasites was similar in both adults (n = 46) and children (n = 41). **B.** In the same group of adults (n = 46) and children (n = 41) as presented in (**A**), IgG binding to 3D7 SBP1KO was markedly reduced compared to 3D7 parental for both groups. **C.** The level of IgG reactivity to the IE surface of E8B parental and E8B SBP1KO parasites was significantly higher in adults (n = 50) compared to children (n = 41). **D.** In the same group of adults (n = 50) and children (n = 41) as presented in (**C**), IgG binding to E8B SBP1KO was substantially reduced compared to E8B parental for both groups. In all graphs, bars represent median and interquartile ranges of samples tested in duplicate; IgG binding levels are expressed as geometric mean fluorescence intensity (MFI) and control refers to serum samples from non-exposed Melbourne residents; assays were performed twice independently. The *p* values in (**A**) and (**C**) were calculated using the Mann–Whitney test and the *p* values in (**B**) and (**D**) were calculated using a paired Wilcoxon signed rank test. (PDF 89 kb)

**Supplemental Figure S3: Antibody levels measured as IgG binding by flow cytometry in pregnant women samples**. A. Serum samples from pregnant women in Malawi tested for antibodies to CS2 parental and CS2 SBP1KO parasites are grouped by gravidity. Samples were from malaria-exposed primigravid (PG; n = 35) and multigravid (MG; n = 36) women and men (n = 10). IgG binding to CS2 SBP1KO parasites was substantially reduced compared to CS2 parental in PG women, MG women and men. There was minimal background reactivity observed among sera from non-exposed Melbourne residents (Control). Assay was performed once; *p* value was calculated using a paired Wilcoxon signed rank test; bars represent median and interquartile ranges of samples tested in duplicate. (PDF 55 kb)

**Supplemental Figure S4: PfEMP1 is the major target of human antibodies**. The overall IgG binding 3D7 SBP1KO was reduced compared to 3D7vpkd and 3D7 parental in PNG (A) and Kenya (C). Assays were performed twice independently; bars represent median and interquartile ranges (n = 81 for PNG, n = 26 for Kenya); *p* value was calculated using a paired Wilcoxon signed rank test; IgG binding levels are expressed as geometric mean fluorescence intensity (MFI) for all graphs. A representative selection of samples tested in parallel for antibodies to 3D7 parental, 3D7vpkd and 3D7 SBP1KO. Samples were from malaria-exposed PNG adults (B; P1-P31), Kenyan adults (D; C12-C25) and non-exposed Melbourne residents (Control). IgG binding to 3D7 SBP1KO was substantially reduced in most individuals, compared to 3D7vpkd and 3D7 parental. There was minimal background reactivity observed among sera from Melbourne residents. Assays were performed twice independently; bars represent mean and range of samples tested in duplicate. (PDF 101 kb)
