# Supplementary figures and images for: A single point in protein trafficking by Plasmodium falciparum determines the expression of major antigens on the surface of infected erythrocytes targeted by human antibodies
Source: Cell Mol Life Sci. 2016 May 19;73(21):4141–58. doi: 10.1007/s00018-016-2267-1 (PMC5042999; doi:10.1007/s00018-016-2267-1)

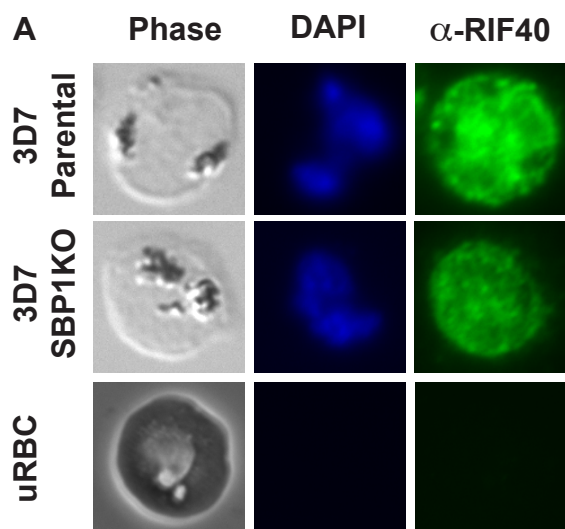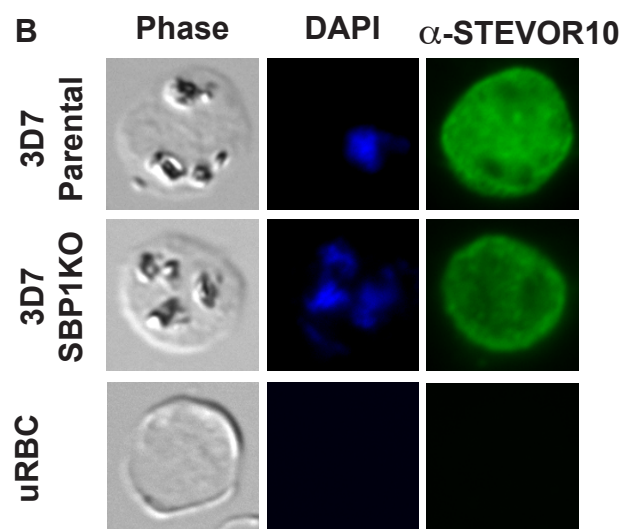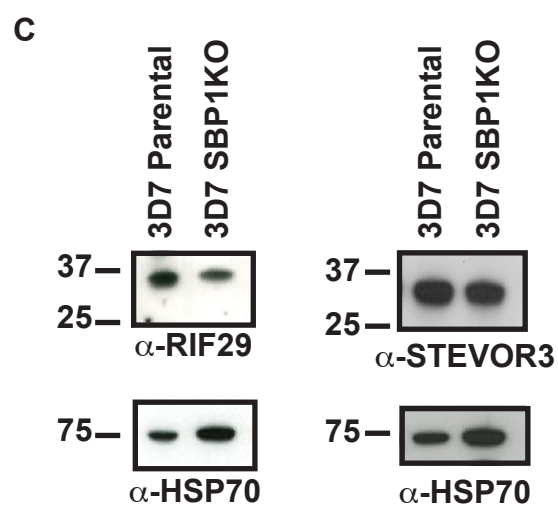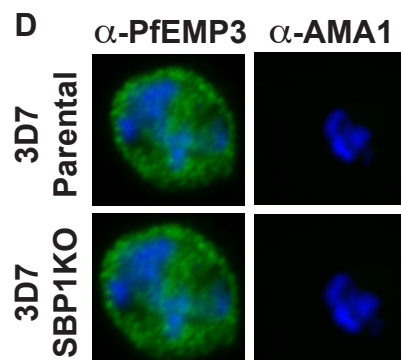

Supplement: Supplementary file 1 — Supplemental Figure S1: Expression of IE membrane proteins by 3D7 parasites. RIFIN (A) and STEVOR (B) proteins expressed by pigmented trophozoite-IEs of 3D7 parental and SBP1KO were visualized by immunofluorescence microscopy using specific α-RIF40 and α-STEVOR10 antibodies (green). RIFIN and STEVOR were detected in 3D7 SBP1KO (middle panel), similar to E8B parental (upper panel). The pattern of staining was consistent with the reported labeling of RIFIN and STEVOR in the IE membrane. The antibodies did not show any labeling of uninfected erythrocytes (uRBC) present in the culture (lower panel).C. Western blot analyses of pigmented trophozoite-IEs membrane extracts probed with α-RIF29 (left) and α-STEVOR3 (right) antibodies, which detected RIFIN at 40 kDa and STEVOR at 30 kDa in both 3D7 parental and 3D7 SBP1KO. Equal loading of parasite material was confirmed with anti-HSP70 antibodies (bottom panel). D. Pigmented trophozoite-IEs of 3D7 parental and 3D7 SBP1KO were probed with α-PfEMP3 antibodies (first column) as a positive control and α-AMA1 antibodies (second column) as a negative control (antibody staining in green, DAPI staining in blue). As expected, the pattern of staining was consistent with the labeling of PfEMP3 in the IE membrane and there was no apparent labeling of AMA1. In all immunofluorescence assays, cells were fixed with a mixture of acetone (90 %) and methanol (10 %) and DAPI was used to stain nuclear DNA (blue). All images were taken at equal exposure for both parasite lines and representative images are shown. (PDF 426 kb) [file 18_2016_2267_MOESM1_ESM.pdf]

A

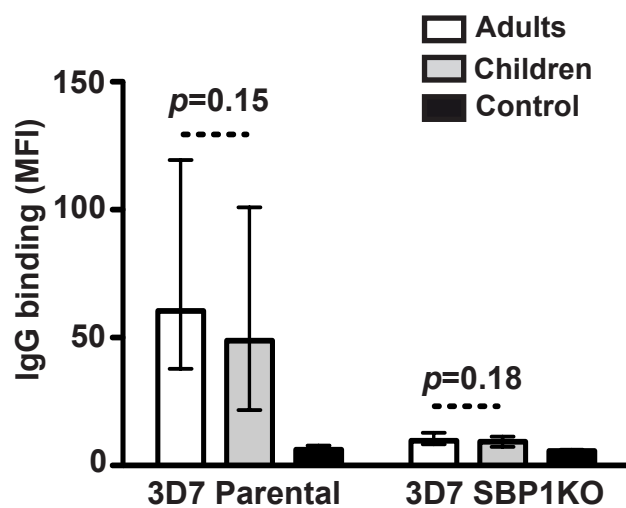

B

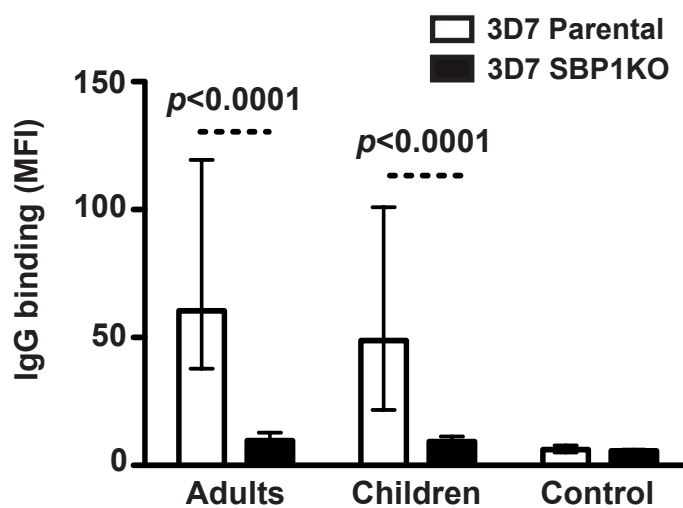

C

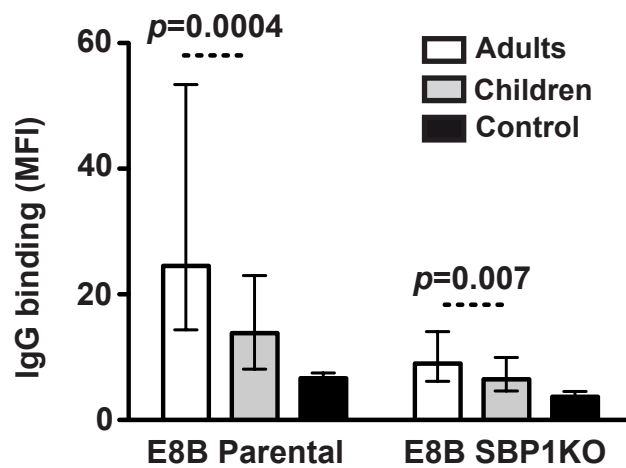

D

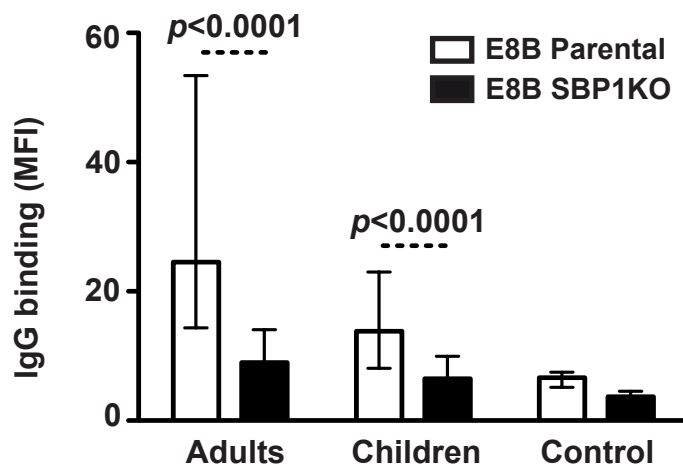

Supplement: Supplementary file 2 — Supplemental Figure S2: Comparing the antibody response between adults and children from PNG. A. The level of IgG reactivity to the IE surface of 3D7 parental and 3D7 SBP1KO parasites was similar in both adults (n = 46) and children (n = 41). B. In the same group of adults (n = 46) and children (n = 41) as presented in (A), IgG binding to 3D7 SBP1KO was markedly reduced compared to 3D7 parental for both groups. C. The level of IgG reactivity to the IE surface of E8B parental and E8B SBP1KO parasites was significantly higher in adults (n = 50) compared to children (n = 41). D. In the same group of adults (n = 50) and children (n = 41) as presented in (C), IgG binding to E8B SBP1KO was substantially reduced compared to E8B parental for both groups. In all graphs, bars represent median and interquartile ranges of samples tested in duplicate; IgG binding levels are expressed as geometric mean fluorescence intensity (MFI) and control refers to serum samples from non-exposed Melbourne residents; assays were performed twice independently. The p values in (A) and (C) were calculated using the Mann–Whitney test and the p values in (B) and (D) were calculated using a paired Wilcoxon signed rank test. (PDF 89 kb) [file 18_2016_2267_MOESM2_ESM.pdf]

A

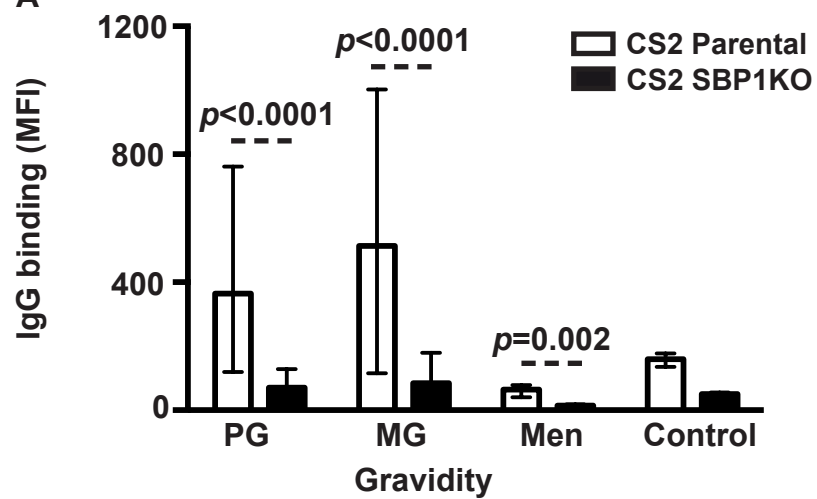

Supplement: Supplementary file 3 — Supplemental Figure S3: Antibody levels measured as IgG binding by flow cytometry in pregnant women samples. A. Serum samples from pregnant women in Malawi tested for antibodies to CS2 parental and CS2 SBP1KO parasites are grouped by gravidity. Samples were from malaria-exposed primigravid (PG; n = 35) and multigravid (MG; n = 36) women and men (n = 10). IgG binding to CS2 SBP1KO parasites was substantially reduced compared to CS2 parental in PG women, MG women and men. There was minimal background reactivity observed among sera from non-exposed Melbourne residents (Control). Assay was performed once; p value was calculated using a paired Wilcoxon signed rank test; bars represent median and interquartile ranges of samples tested in duplicate. (PDF 55 kb) [file 18_2016_2267_MOESM3_ESM.pdf]

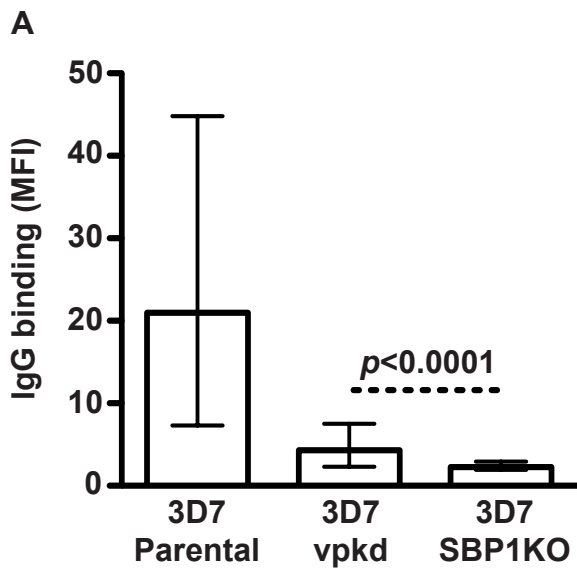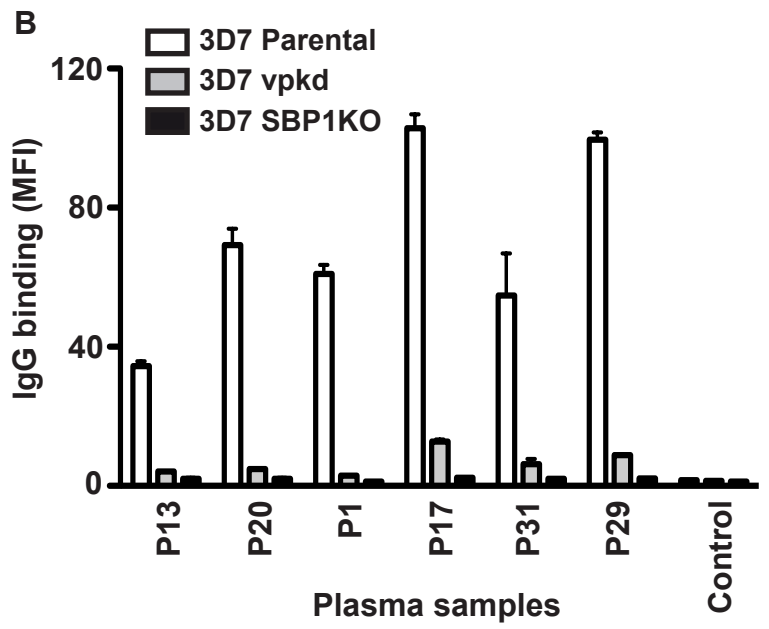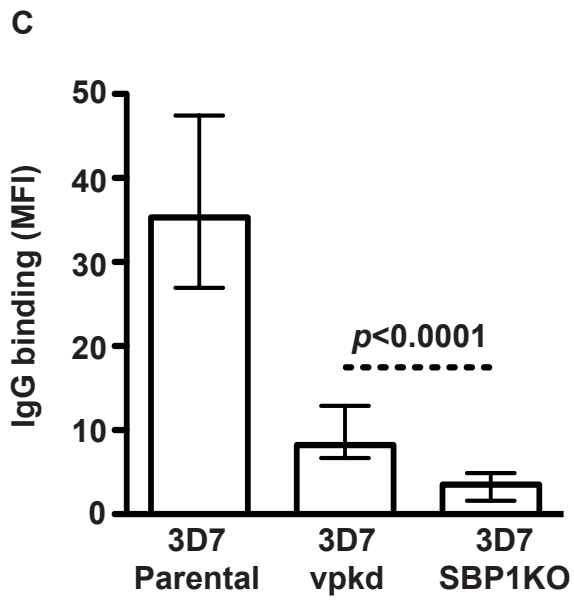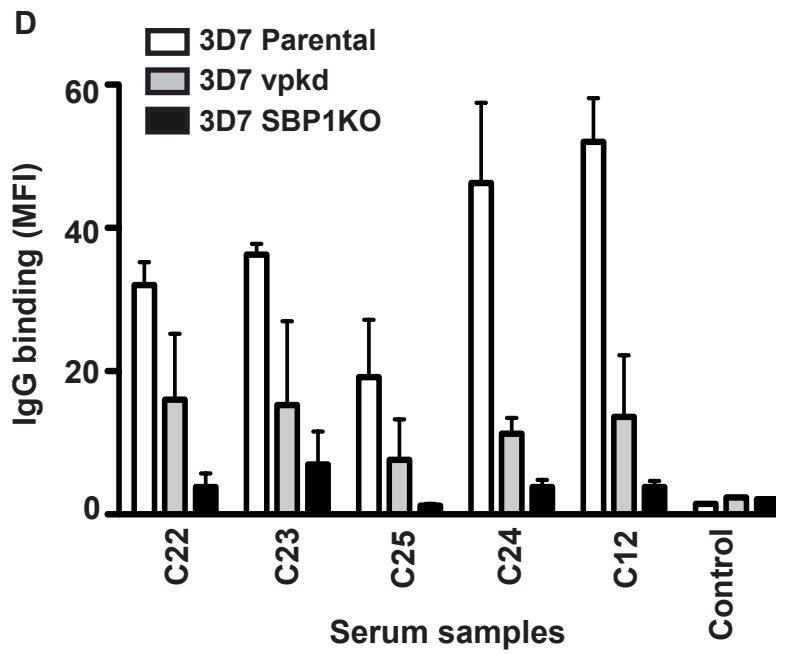

Supplement: Supplementary file 4 — Supplemental Figure S4: PfEMP1 is the major target of human antibodies. The overall IgG binding 3D7 SBP1KO was reduced compared to 3D7vpkd and 3D7 parental in PNG (A) and Kenya (C). Assays were performed twice independently; bars represent median and interquartile ranges (n = 81 for PNG, n = 26 for Kenya); p value was calculated using a paired Wilcoxon signed rank test; IgG binding levels are expressed as geometric mean fluorescence intensity (MFI) for all graphs. A representative selection of samples tested in parallel for antibodies to 3D7 parental, 3D7vpkd and 3D7 SBP1KO. Samples were from malaria-exposed PNG adults (B; P1-P31), Kenyan adults (D; C12-C25) and non-exposed Melbourne residents (Control). IgG binding to 3D7 SBP1KO was substantially reduced in most individuals, compared to 3D7vpkd and 3D7 parental. There was minimal background reactivity observed among sera from Melbourne residents. Assays were performed twice independently; bars represent mean and range of samples tested in duplicate. (PDF 101 kb) [file 18_2016_2267_MOESM4_ESM.pdf]
